# Amphotericin B plus fluorocytosine combined with voriconazole for the treatment of non-HIV and non-transplant-associated cryptococcal meningitis: a retrospective study

**DOI:** 10.1186/s12883-022-02803-1

**Published:** 2022-07-22

**Authors:** Junyu Liu, Jia Liu, Xiaohong Su, Lu Yang, Yijie Wang, Anni Wang, Xiaofeng Xu, Min Li, Ying Jiang, Fuhua Peng

**Affiliations:** Department of Neurology, Center for Mental and Neurological Disorders and Diseases, The Third Affiliated Hospital of Sun Yat-Sen University, Guangzhou, China

**Keywords:** adverse events, cryptococcal meningitis, CSF clearance, Hospitalization time, voriconazole

## Abstract

**Background:**

Our previous study explored Amphotericin B (AMB) plus 5-flucytosine (5-FC) combined with fluconazole (FLU) therapy in the induction period, which seemed to be better than the previous AMB + 5-FC antifungal therapy in non-HIV and non-transplant-associated CM. However, based on our clinical finding, the outcomes of some CM patients who received AMB plus 5-FC combined with FLU antifungal therapy were still poor. Therefore, we need to explore new antifungal methods in non-HIV and non-transplant-associated CM during the induction period.

**Methods:**

Clinical data from 148 patients admitted to the Third Affiliated Hospital of Sun Yat Sen University from January 2011 to December 2020 were collected. These patients were stratified based on antifungal treatment methods in the induction period (group I with AMB + 5-FC + VOR, group II with AMB + 5-FC + FLU, group III with AMB + 5-FC).

**Results:**

The first hospitalization time of Group I (median: 25 days, IQR: 20–34.5) was significantly shorter than that of Group II (median: 43 days, IQR: 29–62) (*p* < 0.001) and Group III (median: 50.5 days, IQR: 43–77.5) (*p* < 0.001). After 2 weeks of follow-up, Group I (26/49) had more patients reaching CSF clearance (*p* = 0.004) than Group II (18/71) and Group III (7/28). In multivariable analysis, Group II (OR: 3.35, 95%CI 1.43–7.82, *p* = 0.005) and Group III (OR: 3.8, 95%CI 1.23–11.81, *p* = 0.021) were associated with higher risk about CSF clearance failure at 2 weeks follow-up than Group I. After 10 weeks of follow-up, the incidence of hypokalemia in Group I was significantly lower than that in Group II (*p* = 0.003) and Group III (*p* = 0.004), and the incidence of gastrointestinal discomfort in Group I was significantly lower than that in Group II (*p* = 0.004).

**Conclusion:**

AMB plus 5-FC combined with VOR may rapidly improve clinical manifestation, decrease CSF OP and clear the cryptococci in CSF during the early phase, substantially shorten the hospitalization time, and reduce the incidences of hypokalemia and gastrointestinal discomfort.

## Background

Cryptococcal meningitis (CM) is a serious disease with high morbidity and mortality [1, 2]. Due to the emergence of highly active antiretroviral therapy (HAART), the morbidity and mortality of HIV-associated CM has gradually decreased [3]. However, the number of CM patients has increased in recent years [4–6]. Recently, some studies have shown that non-HIV CM patients have higher mortality rate than HIV-infected individuals [7, 8]. Therefore, more attention should be paid to the antifungal treatment of non-HIV and non-transplant-associated CM.

Currently, antifungal treatment for CM is divided into three phases: induction therapy, consolidation therapy, and maintenance therapy. Induction therapy is considered to be the most crucial phase of the antifungal treatment for CM. Induction therapy with amphotericin B (AMB) plus 5-flucytosine (5-FC) for at least 4 weeks is recognized as the preferred regimen for non-HIV and non-transplant-associated CM worldwide [9]. However, this regimen is still associated with mortality rates of 15% to 40% [10, 11]. In response to this problem, our previous study explored an AMB plus 5-FC combined with fluconazole (FLU) antifungal therapy in the induction period, which seemed to be better than the previous AMB + 5-FC therapy in non-HIV and non-transplant-associated CM [12]. However, based on our clinical finding, the outcomes of some CM patients who received AMB plus 5-FC combined with fluconazole (FLU) antifungal therapy were still poor. Therefore, we need to explore new antifungal methods in non-HIV and non-transplant-associated CM during the induction period.

Voriconazole (VOR) is a second-generation azole antifungal agent. It is more potent against fungal P-450-dependent 14a-sterol demethylase than FLU and penetrates fungal cells more effectively than FLU [13]. Recently, some case reports showed that VOR could be used as an alternative treatment for CM patients whose standardized treatment failed [14–16]. Therefore, we compared the efficacy of combination therapies among AMB plus 5-FC combined with VOR therapy, AMB plus 5-FC combined with FLU therapy, and AMB plus 5-FC therapy in this study.

## Methods

### Patients and definitions

This study is approved by the Medical Ethics Committee of the Third Affiliated Hospital of Sun Yat-sen University (approval no. [2021] 02–264-01). At admission, the subjects or the guardians of patients with cognitive impairment provided written informed consent for research and publication.

**Fig. 1:**
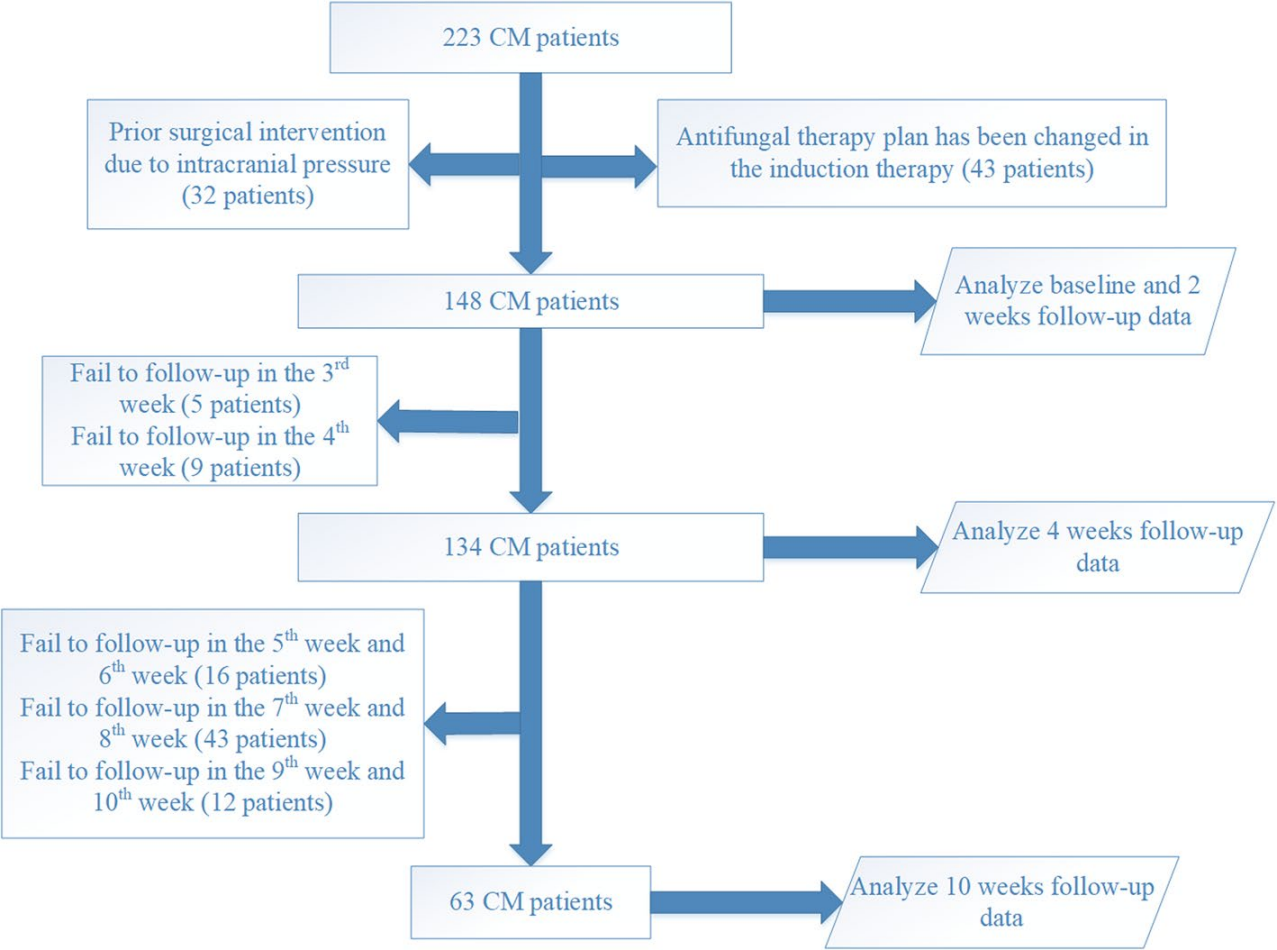
Flow diagram of patient inclusion and exclusion

Data were retrospectively collected from 148 Chinese Han CM patients enrolled between Jan 2011 and Dec 2020 at the Third Affiliated Hospital of Sun Yat-sen University, Guangzhou, China. Medical data were collected from EMRs included demographic data, daily documentation, laboratory results, medications, interventions and diagnosis [17]. CM patients were defined with clinical symptoms, and a positive results of CSF India ink staining or CSF culture for *Cryptococcus neoformans* [18]. Patients meeting all the following criteria were included: (1) CM was firstly diagnosed in our hospital; (2) the antifungal regimen in the induction period was AMB + 5-FC, AMB + 5-FC + FLU, or AMB + 5-FC + VOR (note: AMB used in this study was amphotericin B deoxycholate); and (3) induction therapy was administered for more than 2 weeks. Patients meeting any of the following criteria were excluded: (1) the antifungal regimen was changed in the induction period; (2) a surgical intervention had previously been performed and (3) recurrent CM. A total of 148 non-HIV and non-transplant-associated CM patients was recruited and further divided into three groups according to antifungal treatment methods (49 patients in Group I with AMB + 5-FC + VOR, 71 patients in Group II with AMB + 5-FC + FLU, and 28 patients in Group III with AMB + 5-FC). Due to the loss of follow-up, we obtained the data of all these patients until the loss of follow-up. And the details of the inclusion and exclusion of patients are shown in Fig. 1.

### Clinical manifestation assessment

The British Medical Research Council (BMRC) staging system is an appropriate method to assess the severity of central nervous system (CNS) symptoms, which is divided into three stages: stage 1, normal sensorium with no focal neurological deficit; stage 2, slight or no neurological deficit (cranial nerve palsy) and/or mild clouding of the sensorium; and stage 3, severe focal neurological deficit (multiple cranial nerve palsies), severe impairment of sensorium, convulsions, and/or involuntary movement [19]. The assessment was performed at baseline and after 2, 4 and 10 weeks of antifungal treatment.

### Laboratory examinations

As in our previous study [12], The enrolled patients underwent lumbar punctures at least once a week routinely. CSF opening pressure (OP), CSF white blood cell (WBC), glucose, chloride, protein levels, and CSF cryptococcal counts in India ink staining were recorded.

### Treatment strategies

The treatment strategy followed the 2010 Guideline of the Infectious Diseases Society of America and was based on our previous study and clinical experience [9, 12]. Induction therapy lasted for 2–12 weeks in non-HIV patients, including AMB (See Table 1 for dosages of different groups) and 5-FC (See Table 1 for dosages of different groups), while some of them received FLU (400–800 mg/day) or VOR (loading dose of 6.0 mg/kg/12 h, maintenance dose of 4.0 mg/kg/12 h). Consolidation therapy was given for 8 weeks, including FLU (400–600 mg/day) or VOR (200 mg/12 h). Maintenance therapy lasted for more than 6 months, including FLU (200 mg/day) or VOR (200 mg/day). Mannitol, repeated lumbar puncture, or shunt surgery (ventriculoperitoneal shunt and external drainage of lumbar cistern) were applied to control high CSF pressure.

**Table 1:** Therapeutic regimens in three groups

Variables	Group I (*n* = 49)	Group II(*n* = 71)	Group III(*n* = 28)	*P*-value
The average daily dosages of AMB, mean ± SD, mg/kg/d	0.64 ± 0.14	0.60 ± 0.13	0.55 ± 0.15	0.023*
The lengths of AMB, median (IQR), days	22(16.5–32)	31(23–45)	42(25.5–65.5)	< 0.001*
The total dosages of AMB, median (IQR), mg	705(530–995)	740(543–1359)	985(577–2080.25)	0.088
The average daily dosages of 5-FC, mean ± SD, mg/kg/d	91.41 ± 15.21	86.41 ± 24	80.16 ± 24.97	0.026*
The lengths of 5-FC, median (IQR), days	24(18.5–33)	38(27–60)	47.5(34.25–73.25)	< 0.001*
The total dosages of 5-FC, median (IQR), mg	108,000(92,200–165,600)	160,000(111,000–237,600)	212,250(154,125–270,000)	< 0.001*
the average daily dosages of VOR or FLU, median (IQR), mg/d	400(400–400)	600(400–600)	NA	NA
The length of VOR or FLU median (IQR), days	24(20–33)	33(23–44)	NA	NA
The average daily dosages of mannitol (IQR), ml	277.21(200–342.61)	350(245.96–445.65)	367.90(293.80–474.76)	< 0.001*
The length of mannitol, median (IQR), days	8(3–17)	28(9–44)	46(27–72)	< 0.001*
The average daily dosages of potassium supplementation, median (IQR), g/d	5.02(3.44–6.38)	6.1(4.25–7.26)	5.94(4.73–7.09)	0.077
The average daily dosages of fluid supplementation, mean ± SD, ml/d	1532.13 ± 459.11	1662.92 ± 477.35	1416.70 ± 529.24	0.059

Group I: AMB + 5-FC + VOR, Group II: AMB + 5-FC + FLU, Group III: AMB + 5-FC. NA: Not available. Data were presented as the mean ± SD, median (IQR). Continuous variables were analyzed by one-way ANOVA or Kruskal–Wallis H test

^*^P < 0.05

### Outcome assessments

The treatment response of each patient at the 10th week after the initiation of antifungal therapy was recorded. The therapeutic outcomes were classified into five levels: (1) complete response: survival and resolution of all attributable symptoms and signs of disease with CSF clearance; (2) partial response: survival and CSF clearance with the persistence of attributable symptoms and signs of disease; (3) stable response: survival with minor or no improvement in attributable symptoms and signs of disease and persistently positive CSF culture results; (4) disease progression: worsening clinical disease symptoms or signs and persistently positive CSF culture results; and (5) death: death during the prespecified evaluation period, regardless of cause [20]. CSF clearance was defined as negative CSF cryptococcal culture and CSF cryptococcal organisms count through India ink stain [21].

### Statistical analysis

Baseline demographic and clinical characteristics are presented as percentages, means with standard deviations (SD), or medians with inter-quartile range (IQR). Comparisons were performed using chi-square tests or Fisher's exact tests for categorical data and one-way ANOVA or Kruskal–Wallis H tests for continuous data. The efficacy of treatment was estimated using the chi-square test based on the five levels at the 10th week among the three groups. Chi-square tests were used to compare CSF sterility within 2, 4 and 10 weeks. Chi-square and Fisher's exact tests were used to compare the incidences of adverse events among the three groups. Linear regression was used to fit the distribution of log_10_(CSF cryptococci + 1) at baseline and 2 weeks of follow-up in each treatment regimen group. Binary logistic regression was used to do multivariate analysis about CSF clearance failure at 2 weeks follow-up. And sample calculation about multivariate analysis about CSF clearance failure at 2 weeks follow-up accorded with “Events Per Variable (EPV) more than 10” rules [22]. Statistical analyses were performed using SPSS statistics version 25 (IBM) and R software (version 3.6.2). All analyses were two-sided, and *P*-values < 0.05 were considered statistically significant.

## Results

### Therapeutic regimens

The details of the therapeutic regimens in the three groups are presented in Table 1. AMB, 5-FC, VOR, or FLU were administered by intravenous drip every day for at least 2 weeks.

The average daily dosages of AMB in the different groups were significantly different (*p* = 0.023). By pairwise comparison, the average daily dosages of AMB in Group I (0.64 ± 0.14 mg/kg/day) were more than Group III (0.55 ± 0.15 mg/kg/day) (*p* = 0.022). The length of AMB in the different groups were significantly different (*p* < 0.001). By pairwise comparison, the length of AMB in Group I (median: 22 days, IQR: 16.5–32) was shorter than Group II (median: 31 days, IQR: 23–45) and Group III (median: 42 days, IQR: 25.5–65.5) (p1(Group I VS Group II) < 0.001, p2 (Group I VS Group III) < 0.001). The total dosages of AMB among the three groups were not significantly different (*p* = 0.088).

The average daily dosages of 5-FC in the different groups were significantly different (*p* = 0.026). By pairwise comparison, the average daily dosages of 5-FC in Group I (91.41 ± 15.21 mg/kg/day) were more than Group III (80.16 ± 24.97 mg/kg/day) (*p* = 0.029). The length of 5-FC in the different groups were significantly different (*p* < 0.001). By pairwise comparison, the length of 5-FC in Group I (median: 24 days, IQR: 18.5–33) was shorter than Group II (median: 38 days, IQR: 27–60) and Group III (median: 47.5 days, IQR: 34.25–73.25) (p1 (Group I VS Group II) < 0.001, p2 (Group I VS Group III) < 0.001). The total dosages of 5-FC among the three groups were significantly different (*p* < 0.001). By pairwise comparison, the total dosages of 5-FC in Group I (median: 108,000 mg, IQR: 92,200–165,600) was less than Group II (median: 160,000 mg, IQR: 111,000–237,600) and Group III (median: 212,250 mg, IQR: 154,125–270,000) (p1 (Group I VS Group II) = 0.002, p2 (Group I VS Group III) < 0.001).

The average daily dosage of mannitol among the three groups was significantly different (*p* < 0.001). By pairwise comparison, the average daily dosages of mannitol in Group I (median: 277.21 ml, IQR: 200–342.61) was less than Group II (median: 350.00 ml, IQR: 245.96–445.65) and Group III (median: 367.90 ml, IQR: 293.80–474.76) (p1 (Group I VS Group II) = 0.001, p2 (Group I VS Group III) = 0.001). And the length of mannitol among the three groups was significantly different (p < 0.001). By pairwise comparison, the length of mannitol in Group I (median: 8 days, IQR: 111000–237600) was less than Group II (median: 28 days, IQR: 9–44) and Group III (median: 46 days, IQR: 27–72) (p1 (Group I VS Group II) < 0.001, p2 (Group I VS Group III) < 0.001).

About the average daily dosage of potassium supplementation, there was no significantly difference among the three groups (*p* > 0.05).

### Baseline patient characteristics

The details of the baseline characteristics of patients are presented in Table 2. The first hospitalization time in Group I (median: 25 days, IQR: 20–34.5) was significantly shorter than that in Group II (median: 43 days, IQR: 29–62) (*p* < 0.000) and Group III (median: 50.5 days, IQR: 43–77.5) (*p* < 0.001). However, the hospitalization time between Group II and Group III was not significantly different (*p* = 0.238). There were no significant differences among three groups in other baseline characteristics.

**Table 2:** Baseline characteristics of patients

Variables	Group I (*n* = 49)	Group II(*n* = 71)	Group III(*n* = 28)	*P*-value
Age, mean ± SD, years	48.20 ± 16.44	44.58 ± 14.48	46.00 ± 11.78	0.417
Gender, male (n, %)	32(65.3%)	47(66.2%)	23(82.1%)	0.243
Weight, mean ± SD, Kg	60.596 ± 11.1665	57.757 ± 10.9580	60.346 ± 11.2328	0.340
Length of first hospital stay days, median (IQR), days	25(20–34.5)	43(29–62)	50.5(43–77.5)	< 0.001*
Admission time (n, %), years				< 0.001*
2011–2015	0(0%)	24(33.8%)	21(75.0%)	
2016–2020	49(100%)	47(66.2%)	7(25.0%)	
Underlying diseases (n, %)				
autoimmune disorder	7(14.3%)	6(8.5%)	0(0%)	0.104
immunosuppressive agent	8(16.3%)	7(9.9%)	1(3.6%)	0.213
diabetes mellitus	6(12.2%)	5(7.0%)	0(0%)	0.135
liver cirrhosis	0(0%)	2(2.8%)	1(3.6%)	0.428
Altered mental state (n, %)	5(10.2%)	7(9.9%)	6(21.4%)	0.257
BMRC stage (n, %)				0.632
1	0(0%)	2(2.8%)	0(0%)	
2	42(85.7%)	60(85.4%)	22(78.6%)	
3	7(14.3%)	9(12.7%)	6(21.4%)	
CSF opening pressure, median (IQR), mmH_2_O	240(147.5–330)	266(175–330)	280(162.75–330)	0.311
CSF opening pressure > = 200 (n, %)	31(63.3%)	51(71.8%)	18(64.3%)	0.565
CSF cryptococci, median (IQR), count/ml	56(0–5718)	1296(56–5376)	505(17.25–4308.75)	0.254
CSF white blood cell, median (IQR), *10^6/l	120(47.5–185)	120(64–190)	106.5(34.5–241.5)	0.671
CSF protein, median (IQR), g/l	0.817(0.565–1.362)	0.818(0.56–1.25)	0.655(0.4125–0.9825)	0.133
CSF glucose, median (IQR), mmol/l	1.86(0.73–2.775)	1.33(0.71–2.31)	1.935(1.0825–2.665)	0.366
CSF chloride, median (IQR), mmol/l	116.8(110.8–121.15)	116(109.4–119.3)	117.5(113.35–120.95)	0.158

Group I: AMB + 5-FC + VOR, Group II: AMB + 5-FC + FLU, Group III: AMB + 5-FC. NA: Not available. Data were presented as the mean ± SD, median (IQR). Continuous variables were analyzed by one-way ANOVA or Kruskal–Wallis H test. Categorical variables were analyzed by Chi-square test or Fisher’s exact test

^*^P < 0.05

### Patient characteristics after 2 weeks follow-up

The details of patient characteristics at 2 weeks of follow-up are presented in Table 3. There were significant differences in BMRC stages among three groups (*p* < 0.001), Group I (16/49) had more patients reaching BMRC stage 1 than Group II (7/71) and Group III (0/28) (*p* < 0.001).

**Table 3:** The characteristics of patients after 2 weeks follow-up

Variables	Group I (*n* = 49)	Group II(*n* = 71)	Group III(*n* = 28)	P-value
BMRC stage (n, %)				< 0.001
1	16(32.7%)	7(9.9%)	0(0%)	
2	32(65.3%)	60(84.5%)	27(96.4%)	
3	1(2.0%)	4(5.6%)	1(3.6%)	
Number of lumbar punctures within 2 weeks, median (IQR)	3(3–4)	3(2–4)	3(2–4)	0.431
CSF opening pressure, median (IQR), mmH_2_O	177.5(123.75–220)	210(160–265)	257(220–327.5)	< 0.001
CSF cryptococci, median (IQR), count/ml	0(0–400)	85(0–1500)	72(1–333)	0.061
CSF clearance				
Case (n, %)	26(53.1%)	18(25.4%)	7(25.0%)	0.004*
CSF white blood cell, median (IQR), *10^6/l	70(32–120)	81(28–124)	80(30–123.25)	0.946
CSF protein, median (IQR), g/l	0.955(0.482–2.088)	0.97(0.56–1.80)	0.81(0.4925–1.3325)	0.414
CSF glucose, median (IQR), g/l	2.205(1.015–3.58)	1.83(0.89–2.46)	1.86(1.42–2.215)	0.072
CSF chloride, median (IQR), mmol/l	121.85(115.15–124.60)	118.9(114.2–122.6)	120.05(113.325–124.6)	0.276

Group I: AMB + 5-FC + VOR, Group II: AMB + 5-FC + FLU, Group III: AMB + 5-FC. Data were presented as the median (IQR). Continuous variables were analyzed by one-way ANOVA or Kruskal–Wallis H test. Categorical variables were analyzed by Chi-square test or Fisher’s exact test

^*^P < 0.05

About CSF data, there were no significant differences in the number of lumbar punctures in the three groups within 2 weeks (*p* = 0.431). CSF OP among the three groups was significantly different (*p* < 0.001). By pairwise comparison, CSF OP of Group III (median: 257 mmH_2_O, IQR: 220–327.5) was higher than that of Group I (median: 177.5 mmH_2_O, IQR: 123.75–220) (*p* < 0.001) and Group II (median: 210 mmH_2_O, IQR: 160–265) (*p* = 0.002). CSF clearance among the three groups was significantly different (*p* = 0.004). Group I (26/49) had more patients reaching CSF clearance than Group II (18/71) and Group III (7/28). However, there was not statistically different in CSF WBC, protein, glucose and chloride (*p* > 0.05).

### The decline rate of CSF cryptococci within 2 weeks follow-up

**Fig. 2:**
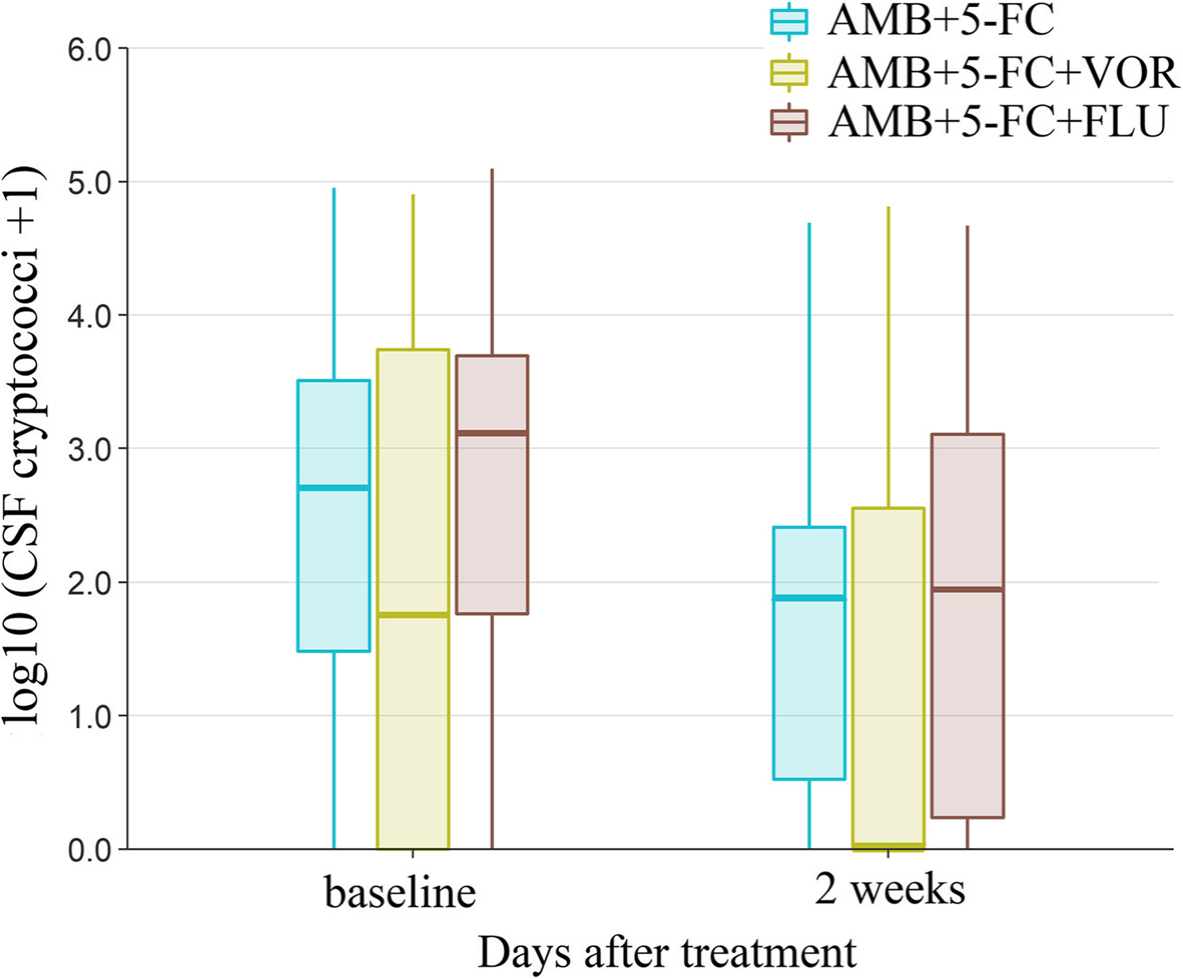
The decline rate of CSF cryptococci within 2 weeks follow-up

The decline rate of CSF cryptococci within 2 weeks follow-up was shown in Fig. 2. The slope in Group I, Group II and Group III was -0.7714, -0.7350 and -0.6296, respectively.

### Multivariable analysis about CSF clearance failure at 2 weeks follow-up

As CSF clearance at 2 weeks follow-up among the three groups was significantly different by univariate analysis, we further did multivariate analysis about CSF clearance failure at 2 weeks follow-up. And the results are presented in Table 4. Interestingly, compared with Group I, Group II (OR: 3.35, 95%CI 1.43–7.82, *p* = 0.005) and Group III (OR: 3.8, 95%CI 1.23–11.81, *p* = 0.021) were independently associated with higher risk about CSF clearance failure at 2 weeks follow-up.

**Table 4:** Multivariate analysis of factors associated with CSF clearance failure at 2 weeks follow-up in non-HIV and non-transplant-associated CM patients

Variable	Univariate^a^		Multivariate^b^	
	OR (95% CI)	*p*-value	OR (95% CI)	*p*-value
Group (ref: Group I)	NA	NA	NA	NA
Group II	3.33 (1.53,7.23)	0.002*	3.35 (1.43,7.82)	0.005*
Group III	3.39 (1.22,9.43)	0.019*	3.80 (1.23,11.81)	0.021*
Autoimmune disorder	1.20 (0.35,4.11)	0.770	0 (0, Inf)	0.999
Immunosuppressive agent	1.66 (0.51,5.43)	0.403	17,144,397.01 (0, Inf)	0.999
Diabetes mellitus	0.61 (0.18,2.09)	0.429	0.44 (0.08,2.47)	0.353
Liver cirrhosis	3,123,919.74 (0, Inf)	0.986	10,359,331.41 (0, Inf)	0.999
Number of lumbar punctures within 2 weeks	1.22 (0.91,1.64)	0.180	1.32 (0.89,1.96)	0.168
Baseline CSF OP	1.00 (1.00,1.01)	0.009*	1.00 (1.00,1.01)	0.118
Baseline CSF cryptococci	1.00 (1.00,1.00)	0.038*	1.00 (1.00,1.00)	0.078

Group I: AMB + 5-FC + VOR, Group II: AMB + 5-FC + FLU, Group III: AMB + 5-FC. NA: Not available

^a^Logistic regression analysis. p-values obtained are 2-tailed

^b^Final logistic regression model containing the covariates with p < 0.2 from the univariable models and some important covariates (autoimmune disorder, immunosuppressive agent, diabetes mellitus, liver cirrhosis) associated with outcome as previous literature reported

^*^p < 0.05

### Patient characteristics after 4 weeks follow-up

The details of patient characteristics at 4 weeks of follow-up are presented in Table 5. There were no significant differences in BMRC stages among three groups (*p* = 0.073).

**Table 5:** The characteristics of patients after 4 weeks follow-up

Variables	Group I (*n* = 41)	Group II(*n* = 67)	Group III(*n* = 26)	*P*-value
BMRC stage (n, %)				0.073
1	17(41.5%)	12(17.9%)	5(19.2%)	
2	23(56.1%)	52(77.6%)	20(76.9%)	
3	1(2.4%)	3(4.5%)	1(3.8%)	
Number of lumbar punctures within 4 weeks, median (IQR)	5(3.5–5.5)	5(4–6)	5(4–5)	0.808
CSF opening pressure, median (IQR), mmH_2_O	162.5(119–211.25)	210(145–260)	240(170–230)	0.006*
CSF cryptococci, median (IQR), count/ml	38.5(0–179.5)	1(0–357)	3(0–56)	0.518
CSF clearance				
Case (n, %)	25(61.0%)	36(53.7%)	7(26.9%)	0.02*
CSF white blood cell, median (IQR), *10^6/l	43.5(18.5–78.25)	50(22–86)	64(16–90)	0.734
CSF protein, median (IQR), g/l	0.97(0.40–1.36)	0.80(0.47–2.27)	0.87(0.42–1.06)	0.446
CSF glucose, median (IQR), g/l	2.25(0.87–3.55)	2.12(1.06–2.64)	2.38(1.76–2.71)	0.456
CSF chloride, median (IQR), mmol/l	122.3(117.4–124.73)	120.8(117.1–123)	122.1(117.1–124.9)	0.395

Group I: AMB + 5-FC + VOR, Group II: AMB + 5-FC + FLU, Group III: AMB + 5-FC. NA: Not available. Data were presented as the mean ± SD, median (IQR). Continuous variables were analyzed by one-way ANOVA or Kruskal–Wallis H test. Categorical variables were analyzed by Chi-square test or Fisher’s exact test

^*^P < 0.05

About CSF data, there were no significant differences in the number of lumbar punctures in the three groups within 4 weeks (*p* = 0.808). CSF OP among the three groups was significantly different (*p* = 0.006). By pairwise comparison, CSF OP of Group I (median: 162.5 mmH_2_O, IQR: 119–211.25) was lower than that of Group II (median: 210 mmH_2_O, IQR: 145–260) (*p* = 0.048) and Group III (median: 240 mmH_2_O, IQR: 170–230) (*p* = 0.005). CSF clearance among the three groups was significantly different (*p* = 0.004). Group I (25/41) had more patients reaching CSF clearance than Group III (7/26) (*P* = 0.02). However, there was not statistically different in CSF WBC, protein, glucose and chloride (*p* > 0.05).

### Patient characteristics after 10 weeks follow-up

The details of patient characteristics at 10 weeks of follow-up are presented in Table 6. There were no significant differences in BMRC stages among three groups (*p* = 0.620).

**Table 6:** The characteristics of patients after 10 weeks follow-up

Variables	Group I (*n* = 15)	Group II(*n* = 30)	Group III(*n* = 18)	*P*-value
BMRC stage (n, %)				0.620
1	9(60.0%)	14(46.7%)	12(66.7%)	
2	6(40.0%)	14(46.7%)	6(33.3%)	
3	0(0%)	2(6.7%)	0(0%)	
Number of lumbar punctures within 10 weeks, median (IQR)	6(4–10)	7(6–11.25)	7(6–8.25)	0.143
CSF opening pressure, median (IQR), mmH_2_O	140(115–220)	175(128.75–232.5)	179(135–207.5)	0.437
CSF cryptococci, median (IQR), count/ml	0(0–0)	0(0–0.75)	0(0–3)	0.868
CSF clearance				
Case (n, %)	11(73.3%)	22(73.3%)	13(72.2%)	1.000
CSF white blood cell, median (IQR), *10^6/l	8(3–22)	16(8–48)	19(7.5–36.5)	0.163
CSF protein, median (IQR), g/l	0.42(0.29–0.75)	0.62(0.36–1.31)	0.57(0.35–1.40)	0.310
CSF glucose, median (IQR), g/l	2.63(2.18–3.90)	2.45(2.11–2.85)	2.64(2.07–3.09)	0.627
CSF chloride, median (IQR), mmol/l	122.2(120.4–125)	121.5(117.48–125)	122.45(121.4–126.03)	0.407
Treatment Outcomes (n, %)				
Complete response	8(53.3%)	14(46.7%)	11(61.1%)	0.622
Partial response	3(20.0%)	6(20.0%)	2(11.1%)	0.757
Stable response	4(26.7%)	8(26.7%)	5(27.8%)	0.709
Disease progression	0(0%)	1(3.3%)	0(0%)	1.000
Death	0(0%)	1(3.3%)	0(0%)	1.000
Adverse events (n, %)	15(100%)	30(100%)	18(100%)	NA
Chills and fevers	0(3.4%)	2(6.7%)	2(11.1%)	0.565
Hypokalemia	7(46.7%)	27(90.0%)	17(94.4%)	0.001*
Mild hypokalemia	0(0%)	6(20.0%)	3(16.7%)	0.188
Moderate hypokalemia	6(40.0%)	13(43.3%)	11(61.1%)	0.388
Severe hypokalemia	1(6.7%)	8(26.7%)	3(16.7%)	0.317
Gastrointestinal discomfort	1(6.7%)	15(50.0%)	4(22.2%)	0.008*
Liver impairment	6(40.0%)	22(73.3%)	12(66.7%)	0.088
Renal impairment	11(73.3%)	19(63.3%)	7(38.9%)	0.105
Mild renal impairment	6(40.0%)	8(26.7%)	3(16.7%)	0.308
Moderate renal impairment	4(26.7%)	9(30.0%)	2(11.1%)	0.346
Severe renal impairment	1(6.7%)	2(6.7%)	2(11.1%)	0.844
Anemia	4(26.7%)	12(40.0%)	7(38.9%)	0.661
Phlebitis	1(6.7%)	2(6.7%)	1(5.6%)	1.000
Visual side effects	1(6.7%)	0(0%)	0(0%)	0.238

Group I: AMB + 5-FC + VOR, Group II: AMB + 5-FC + FLU, Group III: AMB + 5-FC. Data were presented as median (IQR). Continuous variables were analyzed by one-way ANOVA or Kruskal–Wallis H test. Categorical variables were analyzed by Chi-square test or Fisher’s exact test. *P < 0.05. Note: there were 6 observed deaths by week 10 of follow-up. 1 patient died at 3rd week of follow-up, 1 patient died at 5th week of follow-up, 2 patients died at 7th week of follow-up, 1 patient died at 8th week of follow-up and 1 patient died at 10th week of follow-up. The 5 patients who died after less than 10 weeks of follow-up were not analyzed at 10 weeks of follow-up. The 10-week mortality in our study was 6/148 (4.1%)

About CSF data, there were no significant differences in the number of lumbar punctures in the three groups within 10 weeks (*p* = 0.143). Moreover, there was not statistically different in CSF OP, CSF cryptococci, CSF clearance, CSF WBC, protein, glucose and chloride (*p* > 0.05).

In terms of treatment response, there was not statistically different among three groups (p > 0.05). About adverse events, most patients had common adverse events (such as chills and fevers, liver impairment, and renal impairment) during the 10 weeks of treatment (see Table 6). The most common adverse events were renal impairment in Group I (11/15, 73.3%) and hypokalemia in Group II (27/30, 90.0%) and Group III (17/18, 94.4%). The incidence of hypokalemia was significantly different among three groups (*p* = 0.001). Group I had a significantly lower incidence of hypokalemia than Group II (*p* = 0.003) and Group III (*p* = 0.004). However, there was not significantly different in the incidence of mild, moderate and severe hypokalemia among three groups (*p* > 0.05) (note: mild hypokalemia: 3 mmol/L <  = K^+^ < 3.5 mmol/L, moderate hypokalemia: 2.5 mmol/L <  = K^+^ < 3 mmol/L, severe hypokalemia: K^+^ < 2.5 mmol/L). In addition, the incidence of gastrointestinal discomfort among three groups was significantly different (*p* = 0.008). By pairwise comparison, Group I had a significantly lower incidence of gastrointestinal discomfort than Group II (*p* = 0.004).

## Discussion

In this study, we found that the AMB + 5-FC + VOR therapy substantially shortened the hospitalization time, rapidly improved clinical manifestation, decreased CSF OP, cleared the cryptococci from the CSF in the early phase and decreased the incidence of adverse events (hypokalemia and gastrointestinal discomfort) associated with antifungal drugs.

The recommended dose of AMB and 5-FC in induction phase for non-HIV and non-transplant CM is AMB (0.7–1.0 mg/kg per day) plus 5-FC (100 mg/kg per day) [9]. However, most Chinese CM patients could not tolerate AMB and 5-FC at the recommended doses during the induction phase [23]. In our study, the dose of AMB and 5-FC in each group is visually lower than the recommended regimen, which is consistent with our previous study and Chinese expert consensus [12, 24].

Recently, a network meta-analysis showed that AmB + 5-FC + Azole was superior to all other induction regimens in HIV-positive CM patients [25]. And our previous study also showed that AMB + 5-FC + FLU therapy was superior to AMB + 5-FC therapy as the induction regimen in non-HIV CM patients [12]. The reasons why we chose the AMB + 5-FC + VOR therapy to replace the AMB + 5-FC + FLU therapy were as follows: firstly, based on our clinical finding, the outcomes of some CM patients who received AMB + 5-FC + FLU therapy were still poor, and secondly, VOR has been proved to be more effective than FLU in vitro and animal models [13, 26–28].

Elevated intracranial pressure (ICP) is an important factor in morbidity and mortality of CM patients [9, 29]. CM patients with persistent high ICP suffer from cranial nerves injury, usually manifested as visual and hearing loss [30]. Therefore, early controlling of ICP is very important for CM patients. A previous study showed that CM patients with higher fungal burden might have higher CSF OP [31]. Early clearance of *cryptococcus* in CSF can reduce CSF OP and improve the outcome of CM patients. Interestingly, our study suggested that the AMB + 5-FC + VOR therapy may rapidly reach CSF clearance in the early phase. The reasons for this phenomenon may be as follows: (1) VOR has been shown to be more potent and effective than FLU [13, 26–28]; (2) the average daily AMB dosages of the AMB + 5-FC + VOR Group were higher than other two groups. Most CM patients in this study discharged with improved clinical manifestation and CSF results, shorter hospitalization time could reflect the advantage of the AMB + 5-FC + VOR therapy.

In the treatment of CM, it is very important to reduce the incidence of adverse events. The reasons for the low incidence of hypokalemia in the AMB + 5-FC + VOR therapy may be as follows: (1) hypokalemia caused by AMB is dose-dependent [32, 33], the total dosages of AMB in the AMB + 5-FC + VOR therapy were lower than that of the other two groups; (2) VOR is reported to cause hyperkalemia [34] and this may inadvertently counteract the potassium loss effect of AMB, thereby reducing the occurrence of hypokalemia. And the lower incidence of gastrointestinal discomfort in the AMB + 5-FC + VOR therapy might be associate with the shorter hospitalization time.

There were some limitations of our study. First, our study was a retrospective study, which meant that it was prone to produce selection bias and recall bias. And there was an observer bias whereby much more patients were admitted from 2016 to 2020 years in Group I (49/49) than Group II (47/71) and Group III (7/28). Because we often chose AMB + 5-FC + VOR antifungal therapy in induction phase when treating CM patients in recent years, this may lead to this observer bias. Second, our data were obtained from a single center. However, our hospital is the major research unit focused on non-HIV CM in China. Especially, a multicenter study with a larger sample size is needed. Third, early fungicidal activity (EFA) based on quantitative cryptococcal culture was not available because of the limitation of laboratory conditions. In addition, our hospital did not carry out cryptococcal antigen (CrAg) titer test before 2018, and there were many missing data, so we did not include it in the analysis.

## Conclusion

AMB plus 5-FC combined with VOR may rapidly improve clinical manifestation, decrease CSF OP and clear the cryptococci in CSF during the early phase, substantially shorten the hospitalization time, and decrease the incidences of hypokalemia and gastrointestinal discomfort.

## Data Availability

The datasets used and analyzed in this study are available from the corresponding author on reasonable request.

## Abbreviations

CM: Cryptococcal meningitis
AMB: Amphotericin B
5-FC: Flucytosine
FLU: Fluconazole
VOR: Voriconazole
CSF: Cerebrospinal fluid
BMRC: British Medical Research Council
CNS: Central nervous system
OP: Opening pressure
WBC: White blood cell
SD: Standard deviations
IQR: Inter-quartile range
EPV: Events Per Variable
ICP: Intracranial pressure
EFA: Early Fungicidal Activity
CrAg: Cryptococcal antigen

## References

[1] Park BJ, Wannemuehler KA, Marston BJ, Govender N, Pappas PG, Chiller TM (2009). Estimation of the current global burden of cryptococcal meningitis among persons living with HIV/AIDS. AIDS.

[2] Dromer F, Mathoulin-Pelissier S, Launay O, Lortholary O (2007). French Cryptococcosis Study G: Determinants of disease presentation and outcome during cryptococcosis: the CryptoA/D study. PLoS Med.

[3] Antinori S, Ridolfo A, Fasan M, Magni C, Galimberti L, Milazzo L, Sollima S, Adorni F, Giuliani G, Galli M (2009). AIDS-associated cryptococcosis: a comparison of epidemiology, clinical features and outcome in the pre- and post-HAART eras. Experience of a single centre in Italy. HIV Med.

[4] Zonios DI, Falloon J, Huang CY, Chaitt D, Bennett JE (2007). Cryptococcosis and idiopathic CD4 lymphocytopenia. Medicine (Baltimore).

[5] Pukkila-Worley R, Mylonakis E (2008). Epidemiology and management of cryptococcal meningitis: developments and challenges. Expert Opin Pharmacother.

[6] Singh N, Dromer F, Perfect JR, Lortholary O (2008). Cryptococcosis in solid organ transplant recipients: current state of the science. Clin Infect Dis.

[7] Motoa G, Pate A, Chastain D, Mann S, Canfield GS, Franco-Paredes C, Henao-Martinez AF (2020). Increased cryptococcal meningitis mortality among HIV negative, non-transplant patients: a single US center cohort study. Ther Adv Infect Dis.

[8] George IA, Spec A, Powderly WG, Santos CAQ (2018). Comparative Epidemiology and Outcomes of Human Immunodeficiency virus (HIV), Non-HIV Non-transplant, and Solid Organ Transplant Associated Cryptococcosis: A Population-Based Study. Clin Infect Dis.

[9] Perfect JR, Dismukes WE, Dromer F, Goldman DL, Graybill JR, Hamill RJ, Harrison TS, Larsen RA, Lortholary O, Nguyen MH (2010). Clinical practice guidelines for the management of cryptococcal disease: 2010 update by the infectious diseases society of america. Clin Infect Dis.

[10] Day JN, Chau TTH, Wolbers M, Mai PP, Dung NT, Mai NH, Phu NH, Nghia HD, Phong ND, Thai CQ (2013). Combination antifungal therapy for cryptococcal meningitis. N Engl J Med.

[11] Molloy SF, Kanyama C, Heyderman RS, Loyse A, Kouanfack C, Chanda D, Mfinanga S, Temfack E, Lakhi S, Lesikari S (2018). Antifungal Combinations for Treatment of Cryptococcal Meningitis in Africa. N Engl J Med.

[12] Xu L, Liu J, Zhang Q, Li M, Liao J, Kuang W, Zhu C, Yi H, Peng F (2018). Triple therapy versus amphotericin B plus flucytosine for the treatment of non-HIV- and non-transplant-associated cryptococcal meningitis: retrospective cohort study. Neurol Res.

[13] Koltin Y, Hitchcock CA (1997). The search for new triazole antifungal agents. Curr Opin Chem Biol.

[14] Shen YZ, Wang JR, Lu HZ (2008). Voriconazole in an infant with cryptococcal meningitis. Chin Med J (Engl).

[15] Chang HH, Lee NY, Ko WC, Lee HC, Yang YH, Wu CJ, Chang CM (2010). Voriconazole inhibition of tacrolimus metabolism in a kidney transplant recipient with fluconazole-resistant cryptococcal meningitis. Int J Infect Dis.

[16] Carbonara S, Regazzi M, Ciraci E, Villani P, Stano F, Cusato M, Heichen M, Monno L (2009). Long-term efficacy and safety of TDM-assisted combination of voriconazole plus efavirenz in an AIDS patient with cryptococcosis and liver cirrhosis. Ann Pharmacother.

[17] Liang H, Tsui BY, Ni H, Valentim CCS, Baxter SL, Liu G, Cai W, Kermany DS, Sun X, Chen J (2019). Evaluation and accurate diagnoses of pediatric diseases using artificial intelligence. Nat Med.

[18] Bahr NC, Boulware DR (2014). Methods of rapid diagnosis for the etiology of meningitis in adults. Biomark Med.

[19] British Medical Research Council. STREPTOMYCIN treatment of tuberculous meningitis. Lancet. 1948;1(6503):582–96. 18911226

[20] Segal BH, Herbrecht R, Stevens DA, Ostrosky-Zeichner L, Sobel J, Viscoli C, Walsh TJ, Maertens J, Patterson TF, Perfect JR (2008). Defining responses to therapy and study outcomes in clinical trials of invasive fungal diseases: Mycoses Study Group and European Organization for Research and Treatment of Cancer consensus criteria. Clin Infect Dis.

[21] Li Z, Liu Y, Chong Y, Li X, Jie Y, Zheng X, Yan Y (2019). Fluconazole plus flucytosine is a good alternative therapy for non-HIV and non-transplant-associated cryptococcal meningitis: A retrospective cohort study. Mycoses.

[22] Pavlou M, Ambler G, Seaman SR, Guttmann O, Elliott P, King M, Omar RZ (2015). How to develop a more accurate risk prediction model when there are few events. BMJ.

[23] Zhu LP, Wu JQ, Xu B, Ou XT, Zhang QQ, Weng XH (2010). Cryptococcal meningitis in non-HIV-infected patients in a Chinese tertiary care hospital, 1997–2007. Med Mycol.

[24] Liu ZY, Wang GQ, Zhu LP, Lyu XJ, Zhang QQ, Yu YS, Zhou ZH, Liu YB, Cai WP, Li RY (2018). Expert consensus on the diagnosis and treatment of cryptococcal meningitis. Zhonghua Nei Ke Za Zhi.

[25] Chen CH, Li H, Chen HM, Chen YM, Chang YJ, Lin PY, Hsu CW, Tseng PT, Lin KH, Tu YK (2021). Efficacy of induction regimens for cryptococcal meningitis in HIV-infected adults: a systematic review and network meta-analysis. Sci Rep.

[26] van Duin D, Cleare W, Zaragoza O, Casadevall A, Nosanchuk JD (2004). Effects of voriconazole on Cryptococcus neoformans. Antimicrob Agents Chemother.

[27] Pfaller MA, Zhang J, Messer SA, Brandt ME, Hajjeh RA, Jessup CJ, Tumberland M, Mbidde EK, Ghannoum MA (1999). In vitro activities of voriconazole, fluconazole, and itraconazole against 566 clinical isolates of Cryptococcus neoformans from the United States and Africa. Antimicrob Agents Chemother.

[28] Serena C, Pastor FJ, Marine M, Rodriguez MM, Guarro J (2007). Efficacy of voriconazole in a murine model of cryptococcal central nervous system infection. J Antimicrob Chemother.

[29] Molloy SF, Ross B, Kanyama C, Mfinanga S, Lesikari S, Heyderman RS, Kalata N, Ellis J, Kouanfack C, Chanda D (2021). Fungal Burden and Raised Intracranial Pressure Are Independently Associated With Visual Loss in Human Immunodeficiency Virus-Associated Cryptococcal Meningitis. Open Forum Infect Dis.

[30] Liu Y, Peng X, Weng W, Zhu J, Cao H, Xie S (2019). Efficacy of ventriculoperitoneal shunting in patients with cryptococcal meningitis with intracranial hypertension. Int J Infect Dis.

[31] Bicanic T, Brouwer AE, Meintjes G, Rebe K, Limmathurotsakul D, Chierakul W, Teparrakkul P, Loyse A, White NJ, Wood R (2009). Relationship of cerebrospinal fluid pressure, fungal burden and outcome in patients with cryptococcal meningitis undergoing serial lumbar punctures. AIDS.

[32] Llanos A, Cieza J, Bernardo J, Echevarria J, Biaggioni I, Sabra R, Branch RA (1991). Effect of salt supplementation on amphotericin B nephrotoxicity. Kidney Int.

[33] Pathak A, Pien FD, Carvalho L (1998). Amphotericin B use in a community hospital, with special emphasis on side effects. Clin Infect Dis.

[34] Choi JY, Cho SG, Jang KS, Kim GH (2020). Voriconazole-induced Severe Hyperkalemia Precipitated by Multiple Drug Interactions. Electrolyte Blood Press.

